# Transcranial Focused Ultrasound Stimulation Improves Neurorehabilitation after Middle Cerebral Artery Occlusion in Mice

**DOI:** 10.14336/AD.2020.0623

**Published:** 2021-02-01

**Authors:** Jixian Wang, Guofeng Li, Lidong Deng, Muyassar Mamtilahun, Lu Jiang, Weibao Qiu, Hairong Zheng, Junfeng Sun, Qing Xie, Guo-Yuan Yang

**Affiliations:** ^1^Department of Rehabilitation, Ruijin Hospital, Shanghai Jiao Tong University School of Medicine, Shanghai 200025, China.; ^2^Med-X Research Institute and School of Biomedical Engineering, Shanghai Jiao Tong University School of Medicine, Shanghai 200025, China.; ^3^Shenzhen Institutes of Advanced Technology Chinese Academy of Sciences, Shenzhen 518055, China.; ^4^School of Information Engineering, Guangdong Medical University, Dongguan 523808, China

**Keywords:** ischemia, microglia, rehabilitation, stimulation, ultrasound

## Abstract

Transcranial focused ultrasound stimulation (tFUS) regulates neural activity in different brain regions in humans and animals. However, the role of ultrasound stimulation in modulating neural activity and promoting neurorehabilitation in the ischemic brain is largely unknown. In the present study, we explored the effect of tFUS on neurological rehabilitation and the underlying mechanism. Adult male ICR mice (n=42) underwent transient middle cerebral artery occlusion. One week after brain ischemia, low frequency (0.5 MHz) tFUS was applied to stimulate the ischemic hemisphere of mice for 7 consecutive days (10 minutes daily). Brain infarct volume, neurobehavioral tests, microglia activation, IL-10 and IL-10R levels were further assessed for up to 14 days. We found that the brain infarct volume was significantly reduced in the tFUS treated mice compared to that in the non-treated mice (*p*<0.05). Similarly, neurological severity scores, elevated body swing test, and corner test improved in the tFUS treated mice (*p*<0.05). We also demonstrated that tFUS resulted in increased M2 microglia in the ischemic brain region. The expression of IL-10R and IL-10 levels were also substantially upregulated (*p*<0.05). We concluded that tFUS served as a unique technique to promote neurorehabilitation after brain ischemia by promoting microglia polarization and further regulating IL-10 signaling in the ischemic brain.

Stroke is the most common cause of permanent disability, which currently affects approximately 33 million stroke survival worldwide. Over 70% of stroke survival have motor or other neurological functional disabilities. Fortunately, studies showed that standard rehabilitation treatment could enable 80% of survivors to relearn how to walk, 90% of survivors to take care of themselves, and 20-66% of survivors to return to work [1]. It is of great social and economic significance to explore a simple and effective rehabilitation method to promote the recovery of neural function after ischemic stroke.

A range of noninvasive neuromodulation techniques are widely used in experimental neuroscience research and in clinical settings. These techniques include transcranial magnetic stimulation (TMS), transcranial direct current stimulation (tDCS), and transcranial alternating current stimulation (tACS), etc. tDCS and tACS modulate neural excitability by changing the membrane potential through a weak electric current. However, TMS induces sufficiently strong electrical currents to directly activate neurons to generate action potentials on the surface of the ipsilateral hemispherical [2, 3]. It was noted that TMS, tDCS and tACS influenced brain activity after the stimulating period, although their biophysical properties differ in terms of the generated current strength and the spatial and temporal stimulation patterns [4, 5]. The physiological after-effect of interventional brain stimulation are largely unknown; however, there is a consensus that brain stimulation induces homeostatic-like plasticity [6] and long-term potentiation (LTP)-like and long-term depression (LTD)-like effects [7], and causes lasting changes in effective connectivity at the level of the brain network [8]. Since neuromodulation is relatively safe, pain-free, and could promote neuronal plasticity, these techniques attracted general clinical interest for the improvement of brain functions in various brain disorders and prompted substantial efforts [9]. The noninvasive neuronal stimulating technique contributed to the increase in our knowledge about the brain by probing the spatiotemporal features of specific neural substrates. However, critical limitations still existed. For example, TMS lacked the spatial specificity and the depth of penetration [10], and tDCS in the brain tissue showed room for the substantial improvement. Recent developed optogenetic technique provides a superior spatial specificity compared to other brain stimulation methods [11]. However, the optogenetic technique requires cell genetic alterations to express light-activated ion channels [12]. An invasive procedure must be used to introduce the light source to the specific brain region [13]. The optogenetic technique is now in the laboratory experimental stage, and it needs time to improve its feasibility until accepted for the clinical application. Development of a method, which can noninvasively stimulate the brain with superior spatial specificity and deeper penetration, is necessary and timely.

Currently, transcranial focused ultrasound stimulation (tFUS) technique is developed and shows excellent promise in the field of neuromodulation [14, 15]. Previous studies showed that tFUS modulated neuronal activity in mice [16, 17], rats [18], rabbits[19], sheep [20], pigs [21], and monkeys [22]. tFUS is a safe and effective method for the transient neuromodulation in the human somatosensory cortex [23-25], visual cortex, and thalamus [26]. Focused tFUS could have a relative narrow target region even in the rodent brain. This could allow to study the role of specific brain regions in specific behaviors and behavioral disorders [27]. Experimental tFUS studies usually use low frequency (generally below 1 MHz) and low intensity pulsed ultrasound. This low intensity tFUS produces mechanical biological effects without obvious thermal effects and tissue damage. tFUS typically utilizes single [28] or multiple ultrasound transducers that are activated time-dependently [29, 30]. tFUS could deliver highly localized acoustic energy to a specific focal location of interest. Using a lower acoustic frequency, tFUS could penetrate through the intact mouse skull in a focal manner [31, 32]. Using a miniature tFUS headgear, the ultrasound probe could attach to the skull of SD rats through an implanted pedestal, allowing the ultrasound transcranial delivered to the motor cortical area in unanesthetized freely-moving rats [33]. Studies demonstrated that tFUS provided several advantages over other neurostimulation techniques. It allows for spatial specificity and superior penetration depth without requiring an invasive surgical procedure or genetic alterations. Previous studies showed that excitatory pulsed ultrasound stimulation of the ischemic core immediately after ischemic brain injury was neuroprotective, and inhibitory pulsed ultrasound stimulation administered as preconditioning before the induction of photothrombotic stroke mitigated ischemic brain injury by increasing the tolerance of the brain [34]. However, using tFUS to treat animals during ischemic stroke has not been studied.

In the present study, we hypothesize that tFUS improves neurobehavioral outcomes in a mouse model of transient middle cerebral artery occlusion (MCAO). We utilized tFUS to treat MCAO mice to explore: 1) whether tFUS reduces brain injury and improves neurological outcomes after ischemic stroke attack; 2) whether tFUS induces microglia to polarize to M2 microglia; and 3) whether IL-10/IL-10R signaling is involved in reducing the inflammatory response.

## MATERIALS AND METHODS

### Experimental design

**Figure 1.**
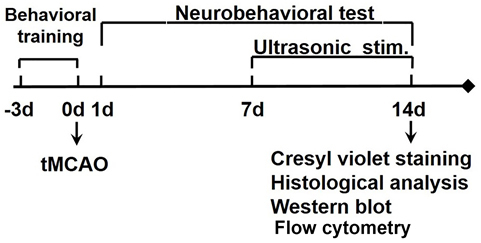
Flow diagram of the experimental design Animals underwent middle cerebral artery occlusion (MCAO). tFUS was applied for 7 days from day 7 of MCAO. The behavioral tests were performed following MCAO for 14 days. Brain atrophy volume and potential mechanisms were examined.

Experimental animal studies were performed in accordance with the Animal Research: Reporting of *in vivo* Experiments (ARRIVE) guidelines with government approval from the State Agency for Nature, the Environment and Consumer Protection North Rhine-Westphalia. The procedure used for the laboratory animals was approved by the Institutional Animal Care and Use Committee (IACUC) of Shanghai Jiao Tong University, China. Animals were housed in cages under standard laboratory conditions. The experimental schedule is shown in Figure 1. Adult male ICR mice underwent 60 minutes of transient MCAO. Occlusion was confirmed by the reduction of cerebral blood flow, as determined by a laser Doppler flowmetry. One week after ischemia, 0.5 MHz low frequency tFUS was applied to the ischemic hemisphere of mice for 7 consecutive days. Brain atrophy volume was calculated via cresyl violet staining. Neurobehavioral outcomes were assessed by neurological severity score (NSS), elevated body swing test (EBST) and corner test. IL-10 and IL-10R were examined to explore the underlying mechanism.

### MCAO procedure

The detailed mice MCAO model was described previously [35, 36]. Adult male ICR mice weighting 26-32 grams (SLAC laboratory animal, Shanghai, China) were anesthetized with ketamine (100 mg/kg) and xylazine (10 mg/kg) intraperitoneally. The body temperature was maintained at 37.0±0.3°C during surgery by a heating pad (RWD life science, Shenzhen, China). A midline incision was made on the neck under an operating microscope (Leica, Wetzlar, Germany). The left common carotid artery (CCA), the external carotid artery (ECA) and the internal carotid artery (ICA) were isolated. A silicone-coated round top 6-0 suture (Dermalon, 1756-31, Covidien, OH) was gently inserted from the ECA stump to the ICA to induce MCA occlusion. The suture inserted distance from the bifurcation to the opening of MCA was 10.5±0.5 mm. The success of occlusion was characterized as the reduction of cerebral blood down to 20%, which was verified by a laser Doppler flowmetry (Moor Instruments, Devon, UK). After 60 minutes of occlusion, the suture was withdrawal, the CCA was restored and blood flow returned to at least 70% of the baseline blood flow. Sham mice underwent the same procedure without inserting the suture.

### Transcranial ultrasound stimulation of the mouse brain after MCAO

**Figure 2.**
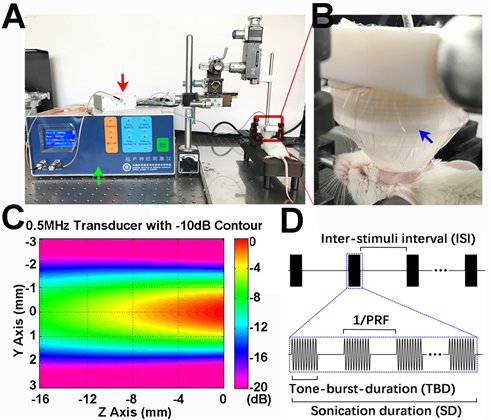
The setup used for low frequency transcranial ultrasound stimulation of the ischemic hemisphere of mice A) Photograph showed the experimental setup of ultrasound stimulation device. B) Photograph showed the tFUS process. The transducer was located on the ipsilateral hemisphere of the mouse brain. C) The photograph showed the ultrasound intensity distribution field relative to the spatial peak in the sanitation region. The Z axis represented the stimulus depth. The Y axis showed the stimulus width. Different colors represented different relative tFUS intensities. Red (high intensity); violet (low intensity). D) The photograph showed a general view of the tFUS sequences: 0.5 ms tone-burst-duration (TBD), 1000 Hz pulse repetition frequency (PRF), 300 ms sonication duration (SD), and 2.7 s inter-stimuli interval (ISI). Green, red and blue arrows indicate the portable tFUS system, the impedance matching device and the ultrasound transducer, respectively.

Mice were divided into three groups: 1) sham group, n=6 for behavioral test; 2) MCAO group, n=18 for neurobehavioral test, infarct volume evaluation, immunostaining, flow cytometry, Western blot and PCR analysis; 3) MCAO/ultra-group, n=18 for behavioral test, infarct volume evaluation, immunostaining, flow cytometry, Western blot and PCR analysis. tFUS experiments were carried out by using a 0.5 MHz focused ultrasound transducer and a portable ultrasound stimulation system [37], which was manufactured by the Shenzhen Institutes of Advanced Technology, Chinese Academy of Sciences. As illustrated in (Fig. 2A, 2B), the transducer was mounted using custom-designed adapters onto the manipulator arms of the stereotaxic frame (Model 68006, RWD Life Science Inc. Shenzhen, China), allowing precise control of the probe on the brain. During the experiment, mice were anesthetized with ketamine/xylazine (100 mg/10 mg/kg body weight). The brain was mounted on the stereotaxic frame. Next, the selected region of the cortex was stimulated with at FUS device. The ultrasound intensity distribution filed relative to the spatial peak in the sanitation region is characterized in Figure 2C. The red region shows a -3 dB intensity area, which was approximately 2 mm in width and 8 mm in depth. Ultrasound stimuli of various intensities with flexible time parameters were generated by the ultrasound transducer, which was powered by the portable ultrasound stimulation system. Figure 2D shows a general view of the stimulation sequences. All of the stimuli parameters could be adjusted in the integrated stimulation system. In this study, the ultrasound stimulation parameters were set as a 0.5 MHz ultrasound frequency, 120 mW/cm^2^ spatial peak pulse average acoustic intensity (Isppa), 0.5 ms of tone burst duration (TBD), 1000 Hz pulse repetition frequency (PRF), 300 ms sonication duration (SD), and 2.7 s inter-stimuli interval (ISI). We chose these parameters because previous studies demonstrated that these parameters were feasible and effective. Seven days following MCAO, ultrasound stimulation was performed for 10 minutes per day over 7 consecutive days.

### Atrophy volume measurement

The brain atrophy volume was measured according to the previous study [38]. Brains were rapidly removed and frozen. The infarct volume was measured using cresyl violet staining. A series of 20 μm frozen coronal sections from anterior commissure to hippocampus were cut. The distance between sections is 200 μm. A total of 20 sections were counted. The infarct area of each section was delineated blindly, and infarct volume was calculated using following formula:

V= 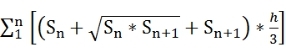, in which, *h* was the distance between two sections. Image analysis software (NIH Image J, Bethesda, MD) was used for the determination of infarct volume.

### Immunostaining

Mice were euthanatized under deep anesthesia. The brains were removed and immersed in 4% paraformaldehyde fixation solution at 4°C overnight. After fixation, the brain was dehydrated in 30% sucrose solution and sectioned in 30 μm thickness. Then brain slices were blocked by 5% normal donkey serum for 60-minutes at room temperature, and then incubated with primary antibodies of IBA1 (1:200, WAKO, Osaka, Japan), CD16/32 (1:100,BD, San Jose, CA), arginase and IL10 receptor (1:100, Santa Cruz, CA) at 4°C overnight, followed by incubation with secondary antibodies (Life Technologies) for one hour at room temperature. The results were observed under a confocal microscopy (Zeiss, Thornwood, NY). The photomicrographs were taken for cell identification.

### Flow Activated Cell Sorter for M1/M2 microglia identification

First, brain cortex was isolated from experimental mice on the ice. Secondly, cell suspension was prepared via gridding brain tissue through 70 μm filters. Then sing cells were centrifuged at 3000 *g* for 10 minutes at 4°C. After centrifugation, we removed the supernatants and washed the cells with 100 μl PBS, followed by centrifugation for 10 minutes at 1000 *g*. Cells were suspended by 100 μl PBS again, and incubated with primary antibodies of CX3CR1-PE (1:200, Biolegend, San Diego, CA), CD16/32-FITC (1:100, Ebioscience, Franklin Lakes, NJ), and CD206-APC (1:50, Biolegend) at room temperature for 15 minutes. These cells were then washed with 3 ml PBS 0.1% sodium azide and 5% fetal bovine serum, followed by centrifugation for 5 minutes at 300 *g* suspend the cells in PBS and analyzed immediately using a Flow Activated Cell Sorter (FACS; BD Bioscience).

### Western blot analysis

Brain tissue containing protein (40 μg) from ipsilateral or contralateral hemisphere were loaded onto 10% resolving gel for electrophoresis. Proteins were transblotted onto a nitrocellulose membrane (Whatman Inc., Florham Park, NJ), and then immuno-probed withIL10 receptor primary antibody (Santa Cruz, CA). The blots were incubated with HRP-conjugated secondary antibody and then reacted with an enhanced chemiluminescence substrate (Pierce, Rockford, IL). The result of chemiluminescence was recorded with an imaging system (Bio-Rad, Hercules, CA).

### RT-PCR assay

First strand cDNA was synthesized from 10 ng RNA using EXQION universal cDNA synthesis kit (EXIQON, Vedbaek, Denmark). The amplification was performed by a fast-real-time PCR system (7900 HT, ABI, Foster City, CA) using a SYBR Green master mix, Universal RT kit (EXQION). A 384-well plate was then running following the cycling condition: 95°C for 10 minutes followed by 40 cycles of 95°C for 10 sec and 60°C for 1 minute. The relative expression level of IL-10 and IL-10R were normalized to the endogenous control GADPH expression in triplicate and were calculated by the 2-Δct method. Primer sequences for each gene were shown as follows:

IL-10 primer sequence:

Forward: 5’ AGGCGCTGTCATCGATTTCT3’

Reverse: 5’ATGGCCTTGTAGACACCTTGG3’

IL-10R primer sequence:

Forward: 5’TTGTCGCGTTTGCTCCCATT3’

Reverse: 5’ GAAGGGCTTGGCAGTTCTG3’

GAPDH primer sequence:

Forward: 5’ AATGGATTTGGACGCATTGGT3’

Reverse: 5’ TTTGCACTGGTACGTGTTGAT3’

### Neurobehavioral assessment

Neurobehavioral assessment was conducted by an experimenter who was blind to the treatment condition. The procedure was used according to previous studies [35]. For neurological function assessment, a modified NSS ranging from 0-14 score was adopted, which included raising the mouse by the tail (0-3), walking on the floor (0-3), beam balance tests (0-6), and the relaxes absence (0-2). For EBST, the mouse tail was lifted to approximately 10 cm above the test platform and the number of times that the mouse body was rotated to the left or right was recorded for a total of 20 times. The percentage of rotation number was determined by one minute at each detection interval. For corner test two flat plates with 30° angle were used to test the behavioral bias of the mice. In the experiment, the mouse was faced between the two plates, and then entered the corner. Both sides of the beard feel the presence of obstacles at the same time, and then turn to the direction of entering the corner. Record the number of turning left or right for total of 20 times. Finally, calculate the deviation index for 1 minute as turn right times/total rotation times.

### Statistical analysis

Data were presented as mean ± SD. Data from Neurological behavior were compared by one-way analysis of variance (ANOVA). Other data were analyzed by student t test using the SPSS software (v18.0, SPSS Inc., Chicago, IL). A probability value of less than 0.05 was considered to represent statistical significance.

## RESULTS

### tFUS improved neurobehavioral outcomes and reduced brain atrophy

**Figure 3.**
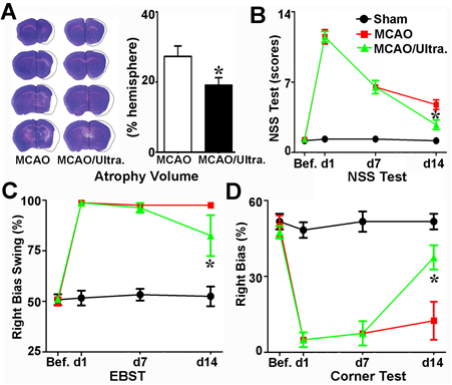
Ultrasound stimulation of the brain promoted neurofunctional recovery in mice that underwent MCAO A) Photomicrographs showed representative sets of cresyl violet-stained brain sections from mice treated with or without tFUS after 60 minutes of MCAO. The line illustrates the atrophy volume of the ipsilateral hemisphere following 7 days of tFUS treatment. Bar graph showed the semi-quantitative data from panel a. n=6 per group, *p*<0.05, tFUS treated mice after MCAO vs. MCAO alone. Line graphs showed the neurologic severity score (B), EBST (C) and corner test (D) results for the sham, MCAO alone, and MCAO mice with tFUS. n=6 per group, *p*<0.05, tFUS treated MCAO mice vs. MCAO alone group.

To evaluate the effect of ultrasound stimulation on therapeutic efficacy, we treated mice with a total of 360 trials of ultrasound stimulation within 10 minutes for 7 consecutive days after 7 days of MCAO. We found that the NSS was greatly increased immediately after MCAO and partially decreased after 7 days of MCAO. The NSS was further decreased in the MCAO-treated mice following 7 days of tFUS but such recovery was not detected in the control mice (*p*<0.05, Fig. 3A). Similarly, the EBST and corner results test were greatly improved in the tFUS-treated mice compared to those in the control mice following 7 days of MCAO (*p*<0.05, Fig. 3B, 3C). This result suggested that tFUS treatment enhanced motor function after ischemic brain injury.

To demonstrate whether the improvement of neurobehavioral outcomes is related to the reduction of ischemia-induced brain atrophy volume after tFUS, we examined brain atrophy volume after 14 days of MCAO. We found that compared to that in the MCAO alone group, the brain atrophy volume was greatly reduced after 7 days of tFUS treatment (*p*<0.05, Fig. 3D). This result suggested that tFUS could attenuate brain tissue loss after ischemic brain injury.

**Figure 4.**
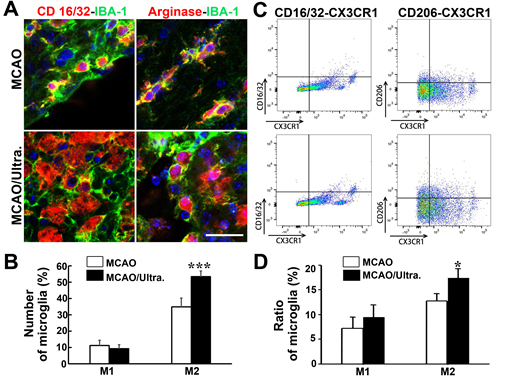
Ultrasound stimulation of the brain increased the number of M2 microglia after MCAO A) Co-immunostaining of the M1 microglia markers CD16/32 and IBA-1 (upper left), and the M2 microglia markers arginase and IBA-1 (upper right) in the mouse brain after one hour of MCAO in mice. After tFUS, there were fewer CD16/32^+^/IBA-1^+^ cells were detected while arginase^+^/IBA-1^+^ cells were increased in the ischemic perifocal region (below). Bar=20 μm. B) Bar graph showed the number of M1 and M2 microglia in the ischemic mouse brain treated with tFUS. Data are presented as mean ± SD. ***, *p<0.001*. C) CD16/32/CX3CR1^+^ (M1) and CD206/CX3CR1^+^ (M2) microglia, the number of CD16/32/CX3CR1^+^and CD206/CX3CR1^+^ microglia were increased. However, the number of CD206/CX3CR1^+^ cells was increased remarkably in the tFUS treated group. D) Bar graph showed the number of M1 and M2 microglia in the ischemic mouse brain treated with tFUS. Data are presented as mean ± SD. *, *p<0.05*.

### tFUS increased the number of M2 microglia

We chose the commonly and typically biomarkers to identify M1 and M2 microglia [39, 40]. To determine whether ultrasound stimulation affects microglia during the post-ischemic recovery period, we performed IBA1^+^/CD16/32^+^ and IBA1^+^/arginase^+^ immunostaining to determine the number of M1and M2 microglia following 14 days of MCAO. The results demonstrated that compared to the control, the number of IBA1^+^/arginase^+^ cells greatly increased in the ipsilateral hemisphere after 7 days of tFUS treated mice (*p*<0.05, Fig. 4A, 4B). This result indicated that tFUS promoted microglia polarization in the ischemia-injured brain.

We then performed FACS to further confirm the effect of tFUS on the changes in microglia. We performed CX3CR1-PE/CD16/32+-FITC and CX3CR1-PE/CD206^+^ -APC immunostaining to identify the number of M1and M2 microglia after 14 days of MCAO. Compared to that in the control group (12.8%), the percentage of CX3CR1/CD206^+^ cells were higher in the tFUS-treated group (17.4%, p<0.05; Fig. 4C, 4D). There was no significant difference in the number of CX3CR1/CD16/32^+^ cells between the experimental (9.5%) and control (7.2%) groups. These results demonstrated that ultrasound stimulation plays an important role in microglia polarization.

### tFUS increased IL-10 and IL-10R expression

**Figure 5.**
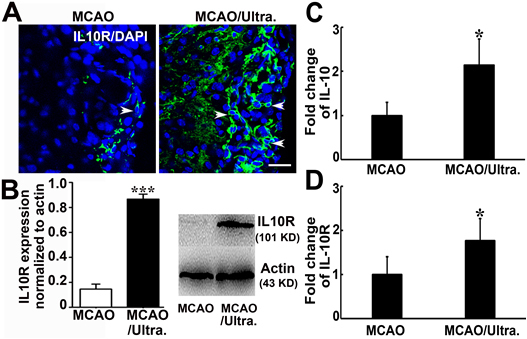
Ultrasound stimulation of the brain upregulated IL-10R and IL-10 A) Photomicrographs showed IL-10R (green) and DAPI (blue) staining in the perifocal region of the ipsilateral hemisphere in sham, MCAO alone, and MCAO mice with tFUS. White arrowheads indicate that IL-10R is mainly expressed in the cell cytosol in the perifocal region in MCAO mice. IL-10R staining was greatly increased after tFUS. Scale bar= 25 μm. B) Western blot analysis showed that IL-10 expression (101 KD) was greatly increased after tFUS. The bar graph showed the quantitative data for the Western blot analysis. n=6 per group. *, *p*<0.05, tFUS treated mice after MCAO vs. MCAO alone. PCR showed the relative expression of IL-10 (C) and IL-10R (D) in the brain after tFUS treatment. n=6 per group. Data are presented as mean ± SD.*, *p<0.05*.

IL-10 is closely related to M2 microglia since they secret IL-10 [41]. In turn, IL-10 could induce microglia to polarize to M2 subtype [42]. Therefore, we speculated that tFUS has an effect on the up-regulation of IL-10/IL-10R signaling. Immunostaining showed that IL-10R was mainly expressed in the cell cytosol in the ipsilateral hemisphere of MCAO mice. IL-10R expression was greatly increased in the tFUS treated MCAO mice compared to that in the control mice (*p*<0.05, Fig. 5A, 5B). We also found that after MCAO, mRNA levels of IL-10 and IL-10R were greatly increased in the tFUS-treated mice. (*p*<0.05, Fig. 5C, 5D)

## DISCUSSION

Using tFUS technique, we demonstrated for the first time that tFUS improved neurobehavioral outcomes and reduced brain atrophy volume in the later stage (7-14 days) after MCAO in mice. The effect of neurorehabilitation was associated with post ischemic microglia polarization, which increased M2 microglia and deceased M1 microglia in the ischemic perifocal areas. Further study demonstrated that IL-10/IL-10R signaling was upregulated. Our results indicated that tFUS provided a unique tool to promote neural repair and remodeling after ischemic stroke. tFUS-based brain stimulation offered several distinct advantages, which allowed better spatial specificity and superior penetration depth without requiring invasive surgical procedure or genetic alteration.

Magnetic, electrical and light isobaric stimulation affect neural plasticity by regulating nerve excitability or promoting neurogenesis. TMS and tDCS stimulation were gradually applied in clinical rehabilitation and showed to promote motor function recovery after stroke [43]. Their application was also extended to the post-stroke aphasia, swallowing problems, and cognitive disorders. Basic research found that these two neuromodulation techniques could promote neurogenesis and synaptic remodeling, and regulate the immune response [44, 45]. Although neuromodulation technique showed its superiority for post-stroke rehabilitation, the existing neuromodulation methods have limitations and weaknesses: for example, pharmacological and chemical methods lack targeting specificity, and electrical stimulation requires the implantation of electrodes. The effect of photogenetics is specific to spatially targeted cells, but it could cause genetic change. TMS and tDCS do not require an invasive operation, but their spatial resolution is only approximately 1 cm, and their depth is generally no more than 2 cm [46]. Efficient neuromodulation techniques may provide a more powerful means for clinical applications. Ultrasound is a type of mechanical acoustic waves. Due to its physical property, acoustic waves can be transmitted over long distances through solid structures including bone and soft tissue. Acoustic waves are widely used in medical and industrial devices [47, 48]. Ultrasound can transmit to tissues as pulse or continuous waveform, which are influenced by heat and/or nonthermal mechanical/biological mechanisms [46]. Compared to chemical, electrical, magnetic or optical methods, the potential of ultrasound for brain stimulation has not being well studied post-stroke rehabilitation. Current studies have shown that ultrasound waves are safer and can reach deeper brain regions to regulate neural plasticity and affect neural activity in humans and animals. Our results demonstrated that tFUS could reduce brain volume atrophy and improve neurobehavioral outcomes, suggesting that the application of ultrasound stimulation in the brain may serve as a novel treatment for clinical stroke rehabilitation.

Microglia play a critical role in the pathological process during ischemic stroke, and are activated, polarized, and converted into classical activated M1 or alternative activated M2 microglia when brain injury occurs. Studies suggested that noninvasive neuromodulation technique could affect microglial function. For example, anodal tDCS modulated the activity and quantity of cortical microglia (IBA1^+^ cells) in ischemic mice. It also caused amoebic morphological changes (M2), which played a role in post-stroke neurorehabilitation [49]. Another experiment found that cathodal tDCS increased the number of M1 type without affecting M2 type microglia [50]. Moreover, low-intensity high-frequency rTMS activated microglia in ischemic rats or global cerebral ischemic gerbils, resulting in increased expression of Iba1 in microglia [51]. In this study, we demonstrated that tFUS increased M2 type microglia following ischemic brain injury, indicating that tFUS treatment promoted microglia polarization into M2 type microglia.

Inflammatory M1 microglia secret inflammatory factors such as TNF-α, iNOS and CCL2 to promote inflammatory processes, which may aggravate autologous brain damage in the early stage of ischemic stroke. M2 microglia secrete anti-inflammatory cytokines such as IL-10. IL-10 is an important immune regulatory factor in the central nervous system. It not only protects brain tissue from inflammation, but also regulates neurogenesis and promotes synaptic remodeling. Studies demonstrated that almost all Nestin^+^ neuro-progenitor cells in the dorsal subependymal region expressed IL-10R. When these neuro-progenitor cells migrated along the rostral side, IL-10 involved in the regulating neurogenesis [52]. Hyperexpression of IL-10 in hippocampal neurons via AAV-IL-10 transduction increased the number of DCX^+^ cells and BrdU^+^/NeuN^+^ cells in the dentate gyrus and consequently improved the spatial memory in mice [53]. When NSC overexpressed IL-10, which transplanted into autoimmune encephalitis mice, they not only inhibited the inflammatory response but also promoted the differentiation of neuronal stem cells into oligodendrocytes and neurons [54]. These studies suggested that IL-10 is a key factor involved in poststroke rehabilitation. In our study, we found that the number of M2 microglia increased after tFUS treatment; it was also noted that IL-10 and IL-10R upregulated, suggesting that tFUS modulated microglia polarization was associated with IL-10/IL-10R signaling.

We concluded that tFUS provided a safe and noninvasive approach to enhance post ischemic stroke rehabilitation by promoting microglia polarization, reducing inflammatory response, and improving neuronal repair and remodeling. It would be a unique tool for the clinical use in humans after extensive safety and efficacy studies.
